# The Effect of Human Immunodeficiency Virus and Cytomegalovirus Infection on Infant Responses to Vaccines: A Review

**DOI:** 10.3389/fimmu.2018.00328

**Published:** 2018-03-02

**Authors:** Olivia Falconer, Marie-Louise Newell, Christine E. Jones

**Affiliations:** ^1^Institute for Life Sciences, Faculty of Medicine, University of Southampton, University Hospital Southampton NHS Foundation Trust, Southampton, United Kingdom; ^2^Institute of Developmental Science, Human Development and Health, Faculty of Medicine, University of Southampton, Southampton, United Kingdom

**Keywords:** human immunodeficiency virus, cytomegalovirus, vaccines, immune responses, infant

## Abstract

The success of prevention of mother to child transmission programs over the last two decades has led to an increasing number of infants who are exposed to human immunodeficiency virus (HIV), but who are not themselves infected (HIV-exposed, uninfected infants). Although the morbidity and mortality among HIV-exposed, uninfected infants is considerably lower than that among HIV-infected infants, they may remain at increased risk of infections in the first 2 years of life compared with their HIV-unexposed peers, especially in the absence of breastfeeding. There is some evidence of immunological differences in HIV-exposed, uninfected infants, which could play a role in susceptibility to infection. Cytomegalovirus (CMV) may contribute to the increased immune activation observed in HIV-exposed, uninfected infants. Infants born to HIV-infected women are at increased risk of congenital CMV infection, as well as early acquisition of postnatal CMV infection. In infants with HIV infection, CMV co-infection in early life is associated with higher morbidity and mortality. This review considers how HIV infection, HIV exposure, and CMV infection affect infant responses to vaccination, and explores possible immunological and other explanations for these findings. HIV-infected infants have lower vaccine-induced antibody concentrations following tetanus, diphtheria, pertussis, hepatitis B, and pneumococcal vaccination, although the clinical relevance of this difference is not known. Despite lower concentrations of maternal-specific antibody at birth, HIV-exposed, uninfected infants respond to vaccination at least as well as their HIV-unexposed uninfected peers. CMV infection leads to an increase in activation and differentiation of the whole T-cell population, but there is limited data on the effects of CMV infection on infant vaccine responses. In light of growing evidence of poor clinical outcomes associated with CMV infection in HIV-exposed, uninfected infants, further studies are particularly important in this group. A clearer understanding of the mechanisms by which maternal viral infections influence the developing infant immune system is critical to the success of maternal and infant vaccination strategies.

## Introduction

Immunization is essential to global strategies to reduce infant mortality, especially in low-resource settings where infectious morbidity and mortality remain high (1, 2). In regions of the world where the burden of infectious diseases is high, even a small reduction in vaccine efficacy might have important clinical implications for young infants. Factors affecting infant vaccine responses can be divided into those relating to the infant, mother and environment; an important and potentially modifiable maternal factor is antenatal viral infections. To date, studies of the effects of maternal antenatal viral infections on infant vaccine responses have focused on two important viral infections: human immunodeficiency virus (HIV) and cytomegalovirus (CMV).

In 2015, there were 1.4 million pregnant women living with HIV (3), and an estimated 24% of those did not receive antiretroviral therapy (ART) for prevention of mother-to-child transmission (PMTCT) (4). In infants born with HIV infection, early commencement of combination ART significantly reduces mortality (5, 6) and is associated with increased magnitude and quality of infant antibody responses to vaccines (7–10), but there is some evidence of reduced humoral responses to vaccines even in those children starting ART at 6–8 weeks of age, compared with HIV-unexposed infants (9).

The expansion of PMTCT programs in the last two decades, and continued high numbers of HIV-infected women who become pregnant, have led to an increase in the number of HIV-exposed, uninfected infants, who now represent up to 30% of births in some parts of Southern Africa (11). Compared with HIV-unexposed infants, HIV-exposed, uninfected infants are at increased risk of hospitalization, pneumonia and mortality at 6 and 24 months in some settings, although the risk is reduced when they are breastfed and when infected children and their mothers receive ART (12). Increasing evidence shows immunological differences in HIV-exposed, uninfected infants, who have changes in T-cell populations, lower CD4 counts and increased T-cell differentiation by 10 weeks of age compared with HIV-unexposed infants (13). While these immunological differences may play a part in the increased morbidity which has been observed, factors such as socioeconomic status, maternal health, breastfeeding duration, exposure to ART, and exposure to co-infections are also likely to be important.

Cytomegalovirus is the most common congenital infection worldwide, affecting up to 1.2% of live births in developing countries. Although CMV infection is thought to lead to immune senescence and a poor response to the influenza vaccine in the elderly (14), it is not fully understood how CMV might affect infant responses to vaccines. Congenital CMV infection leads to changes in the infant T-cell population as a whole and is associated with increased morbidity in HIV-infected and HIV-exposed, uninfected infants. In HIV-infected infants who are not receiving ART, those with congenital CMV infection have an increased rate of HIV disease progression (15, 16). HIV-exposed infants may be at higher risk of congenital CMV than HIV-unexposed infants (17, 18). In HIV-exposed, uninfected infants, CMV infection may contribute to immune activation (11, 19, 20) and increase the risk of postnatal HIV infection (21, 22).

This review summarizes the evidence for alterations in infant vaccine responses associated with exposure to maternal HIV and CMV infection, and explores possible immunological and other explanations for these findings.

## Methods

A literature search of English language publications was performed using Medline. Key search terms included: maternal, fetal, neonate, HIV, CMV, vaccination, and immunization. The full search strategy is detailed in Table S1 in Supplementary Material. A formal systematic review was not undertaken; how-ever, we sought to carry out a comprehensive review of the published literature. We screened titles and abstracts, selecting all articles with outcomes that included infant immune responses to routine Expanded Program on Immunizations vaccinations following antenatal exposure to either maternal HIV or CMV infection, or both.

Studies of HIV-infected infants in which the majority of participants received ART were selected when possible, for three main reasons: (1) ART is associated with increased magnitude and quality of infant antibody responses to vaccines (7–10); (2) studies in which infants do not receive ART are prone to survival bias because there is often a high mortality rate in the HIV-infected group (7, 9, 23); and (3) this review is intended to be relevant to current and future practice, looking toward universal ART in HIV-infected children. Since 2015, the World Health Organization guidelines have recommended that all children infected with HIV receive ART. The proportion of children with HIV receiving ART worldwide was 49% in 2015, and ART coverage in children and adults with HIV has been increasing year on year (24). Studies of vaccine responses in HIV-infected infants were excluded if they did not include a HIV-unexposed control group. Papers including a comparison between vaccine responses in HIV-exposed, uninfected infants with HIV-unexposed, uninfected infants were included. This review aimed to review the contemporary literature; there were no studies comparing vaccine responses of HIV-infected infants treated with ART and HIV-exposed, uninfected infants, therefore this comparison is not made in this review.

## Vaccine Responses in HIV-Infected Infants

### Humoral Responses

The published literature shows some heterogeneity in the differences in concentrations of specific immunoglobulin (Ig) G and proportion of infants protected following primary and booster doses of routine vaccines in HIV-infected infants receiving ART compared with HIV-unexposed infants (Table 1).

**Table 1:** Effect of HIV infection on infant responses to vaccines.

	Vaccine	Age at vaccination	Age at blood sampling	HIV-infected infants on ART, *n*	HIV-unexposed infants, *n*	Main findings in HIV-infected compared with HIV-unexposed infants
South Africa (7)	PCV	7, 11, and 15 weeks	20 weeks	172	125	No significant difference in % protected or concentration of specific IgG Opsonophagocytic assay: higher concentration of antibody needed for 50% killing activity for 2/3 serotypes (antibody (95% CI) for 50% killing 24 (21–27) vs 19 (16–23), *p* = 0.045 for serotype 19F; 4 (3–5) vs 2 (2–3), *p* = 0.007 for serotype 23F)

South Africa (8)	PCV	7, 11, and 15 weeks	7, 11, 15, and 20 weeks	205	119	Pre-vaccination, lower concentration of specific IgG for 3/7 serotypes (mean GMC 0.14 vs 0.22, *p* ≤ 0.004) and lower % protected for 4/7 serotypes (mean 30 vs 45%, *p* ≤ 0.008) Following dose 1, significantly lower specific IgG concentrations for 3/7 serotypes (mean 0.42 vs 0.60, *p* ≤ 0.016) and lower % protected (mean 34 vs 49%, *p* ≤ 0.007) for 4/7 serotypes No difference after dose 2

South Africa (9)	DTwP-Hib, HBV	6, 10, and 14 weeks	7 and 20 weeks	172	114	Pre-vaccination, lower concentration of antibody to tetanus (GMC 0.086 vs 0.421 IU/ml *N*/*n^m^, p* < 0.001), HBsAg (GMC 5.81 vs 7.74 mIU/ml *N*/*n, p* = 0.01), and pertussis (GMC 17.67 vs 40.65 IU/ml, *p* < 0.001) Post-vaccination antibody concentration lower for tetanus (GMC 0.405 vs 0.952 IU/ml *N*/*n^m^, p* < 0.001), diphtheria (GMC 0.200 vs 0.272 IU/ml *N*/*n, p* = 0.001), HBsAg (GMC 924.87 vs 2,521.03 mIU/ml *N*/*n, p* < 0.001), and pertussis (GMC 40.48 vs 61.24 IU/ml, *p* < 0.001), but no difference in % protected

South Africa (10)	Measles	9 and 15.5 months	2.5, 4, 15.5, 16, and 24 months	182	115	Pre-vaccination, significantly lower % protected (41.8 vs 65.2%) At 24 months, no significant difference in antibody concentration or % protected

Zambia (21)	OPV	0, 6, 10, and 14 weeks, 12 months	18 months	17	397	Lower antibody titer (log_2_ antibody titer 4.71 (SD 2.97) vs 8.15 (SD 2.09), *p* < 0.01) Lower proportion had protective antibody levels (64.5 vs 98.4%, *p* < 0.01)

*PCV, pneumococcal conjugate vaccine; DTwP-Hib, diphtheria, tetanus, whole cell pertussis, Hib; OPV, oral polio vaccine; % protected, proportion protected; CI, confidence interval, Ig, immunoglobulin; HBSAg, hepatitis B surface antigen; ART, antiretroviral therapy; HIV, human immunodeficiency virus*.

Following primary immunization, HIV-infected infants aged 20 weeks had significantly lower specific antibody concentrations to tetanus, diphtheria, pertussis and hepatitis B surface antigen (HBSAg), than HIV-unexposed infants in a South African study (9). However, the clinical significance of this is unclear, as high proportions (92–100%) of infants from both groups had concentrations of antibody to tetanus, diphtheria, and HBsAg that are deemed protective (9).

Pneumococcal conjugate vaccine (PCV) antibody concentrations were lower in HIV-infected infants after the first and second PCV doses (8), but after the third dose the antibody concentration and proportion of infants protected were similar in HIV-infected and HIV-unexposed infants (7). However, an op-sonophagocytic assay showed that antibody against two out of three PCV serotypes tested in HIV-infected infants receiving ART had 26–50% lower killing activity than that of HIV-unexposed infants. This suggests that HIV-infected infants may not mount as effective an antibody response against pneumococcal disease as HIV-uninfected infants, despite producing a similar concentration of antibody following vaccination (7). A UK study of older children (mean age 12.8 years, range 1–17.4 years) showed that a lower proportion of HIV-infected individuals were protected against three of 13 PCV serotypes, compared with HIV-unexposed children and adults. This was despite an equal or larger proportion of HIV-infected children having previously received the 7-valent or polysaccharide pneumococcal vaccines (25).

In a Zambian cohort in which mothers received short-course intrapartum nevirapine to prevent mother-to-child transmission during labor and delivery, but infants did not receive ART, HIV-infected infants who received oral polio vaccine (OPV) had 42% lower neutralizing antibody responses at 18 months (*p* < 0.01), and a lower proportion had protective antibody levels than HIV-unexposed infants (64.5 vs 98.4%, *p* < 0.01) (26).

Following measles vaccination at age 9 months, there was no significant difference in measles-specific IgG levels at 24 months in HIV-infected infants compared with HIV-unexposed infants in a study from South Africa, and no difference in the proportion of infants with protective antibody levels (10).

Human immunodeficiency virus-infected newborns are potentially more susceptible to vaccine-preventable diseases for a longer period than HIV-unexposed infants (8, 10). As well as having lower responses to the first two doses of vaccine (as seen with PCV and measles), compared with HIV-unexposed infants, HIV-infected infants had lower pre-vaccination concentrations of antibody to three of seven PCV serotypes (8), tetanus (GMC 0.086 vs 0.421, *p* < 0.001), HBsAg (GMC 5.81 vs 7.74, *p* = 0.01), and pertussis (GMC 17.67 vs 40.65, *p* < 0.001) (9, 10). Similarly, before vaccination a lower proportion of HIV-infected than HIV-unexposed infants had protective levels of antibody to PCV (for four serotypes, mean 30 vs 45% protected, *p* ≤ 0.008) and measles (9, 10).

### Cellular Responses

In a study in South Africa, in which mothers received PMTCT and infected infants (diagnosed at 6 weeks) were not breastfed and did not receive ART, T-cell responses to BCG in HIV-infected infants were compared with those in HIV-unexposed infants (27). After BCG vaccine on day 1 of life, HIV-infected infants had severely impaired T-cell responses at 3 months, and by 9–12 months the response was almost absent (27). Both the magnitude of the CD4 and CD8 T-cell responses, and the polyfunctionality of the CD4 response were markedly reduced (27). Secreted cytokines interferon (IFN)-γ and interleukin-2 were also present in significantly lower concentrations in HIV-infected than HIV-unexposed infants at 3 months, although tumor necrosis factor (TNF)-α concentration was not significantly different (27).

### Mechanisms of Altered Vaccine Responses in HIV-Infected Infants

Both maternal and infant factors are likely to be involved in the observed differences in antibody and cellular responses to vaccines seen in HIV-infected compared with HIV-unexposed infants (8). In HIV-infected infants, lower CD4 count may impair the mechanisms leading to induction and maintenance of immunological memory to vaccine antigens (26). The observation that specific antibody concentrations are lower in HIV-infected infants before vaccination may suggest reduced transfer of antibody across the placenta (8, 10). Both reduced antibody concentration and impaired placental function in HIV-infected mothers may contribute to this (28, 29).

There are inherent difficulties in comparing vaccine responses in HIV-infected with HIV-unexposed infant populations, as the two groups are likely to differ in duration of breastfeeding, exposure to ART, socioeconomic status, exposure to co-infections, nutritional status, and survival. In a multivariable analysis of factors associated with response to OPV (primary course and booster at age 12 months) at 18 months of age, increasing breast-feeding duration was associated with increasing poliovirus antibody level (26). In this study, median breastfeeding duration was 6 months in HIV-infected mother-infant pairs compared with 15 months in HIV-uninfected pairs (*p* < 0.01). Differences in breastfeeding duration in HIV-infected and unexposed groups were not stated in the other studies described earlier, although in four of the studies, infants were co-enrolled in the CHER trial in South Africa, a randomized controlled trial evaluating antiretroviral treatment strategies, in which only 14% of infants were breastfed (30). Short duration or refraining from breastfeeding in low and middle-income settings, including for HIV-infected infants, is associated with increased infectious morbidity, stunting, and wasting (31–33).

A recent review of the effects of maternal nutritional status on infant vaccine responses concluded that maternal macro- and micronutrient deficiency during pregnancy is likely to impair infant responses to vaccines, even in the presence of nutrient supplementation (34).

Increased exposure to opportunistic infections through breastfeeding (for example, CMV infection) or close contact with HIV-infected mothers who may have co-infection may affect immune responses in HIV-infected infants. In HIV and CMV co-infection, infants have accelerated HIV progression, increased mortality, growth delay and cognitive impairment, compared with HIV-infected infants without CMV (35). In Malawi, breastmilk CMV load had a stronger negative association with infant growth than breastmilk HIV load (36).

In summary, HIV-infected infants receiving ART may have impaired ability to mount quantitatively and qualitatively ade-quate antibody responses to vaccines compared with HIV-unexposed infants. The clinical effect of impaired vaccine responses on morbidity from vaccine-preventable disease is not known. Shorter breastfeeding duration, poorer nutritional status and increased exposure to co-infections in HIV-infected infants may be contributing factors, and their impact requires further investigation to fully understand the mechanisms underlying the changes in vaccine responses in HIV-infected infants. CMV infection is almost ubiquitous in low-resource settings, and its clinical effects on infants with HIV suggest it is having an important effect on the immune system, and could be an important modifiable factor in reducing morbidity and mortality of HIV-infected infants.

## Vaccine Responses in HIV-Exposed, Uninfected Infants

### Humoral Responses

Detailed studies in HIV-exposed, uninfected infants have demonstrated differing patterns of antibody responses to vaccines compared with HIV-unexposed infants (Table 2), and revealed likely underlying mechanisms.

**Table 2:** HIV-exposed, uninfected infant responses to vaccines.

	Vaccine	Age at vaccination	Age at blood sampling	HIV-exposed uninfected infants, *n*	HIV-unexposed infants, *n*	Findings in HIV-exposed uninfected compared with HIV-unexposed infants
South Africa (7)	PCV	7, 11, and 15 weeks	20 weeks	120	125	Overall no difference in specific IgG concentration or % protected after third dose (median 99 vs 98% protected)

South Africa (8)	PCV	7, 11, and 15 weeks	7, 11, 15, and 20 weeks	124	119	Pre-vaccination: for 7/7 serotypes, significantly lower specific IgG concentration (median GMC 0.12 vs 0.21, *p* ≤ 0.006) and % protected (median 18 vs 32%, *p* ≤ 0.005) Post-dose 1: significantly lower specific IgG concentration (4/7 serotypes, median GMC 0.26 vs 0.53, *p* ≤ 0.003) and % protected (5/7 serotypes, median 35 vs 49%, *p* ≤ 0.019) Post-dose 2: overall no significant difference in GMT or % protected

South Africa (9)	DTwP-HibCV, HBV	6, 10, and 14 weeks	7 and 20 weeks	120	114	Pre-vaccination: lower antibody concentration against tetanus (GMC 0.219 vs 0.421 IU/ml *N*/*n^m^, p* = 0.001); higher antibody concentration and % protected against diphtheria (61 vs 29% protected, *p* < 0.001) and HBsAg (81 vs 50% protected, *p* < 0.001) Post-vaccination: lower antibody concentration against HBsAg (GMC 2,019.28 vs 2,521.03, *p* = 0.041) but mean 99.2% protected. Higher antibody concentration (GMC 261.30 vs 134.34, *p* < 0.001) and % achieving fourfold increase (76.7 vs 39.1%, *p* < 0.001) in response to pertussis

South Africa (10)	Measles	9 and 15.5 months	2.5, 4, 15.5, 16, and 24 months	116	115	Pre-vaccination: no significant difference in GMT or % protected at 2.5 months Before booster (15.5 months): significantly higher antibody concentration (GMT 3,009 vs 2,212, *p* = 0.008) but no difference in % protected After booster: significantly lower antibody concentration at 16 months (GMT 2,532 vs 3,124, *p* = 0.015) and 24 months (GMT 1,773 vs 2,248, *p* = 0.004), and lower % protected at 24 months (79.6 vs 94.3%, *p* = 0.002)

Malawi (13)	BCG, OPV	Birth	10 weeks	13	21	No difference in anti-*M. tb* and anti-polio IgG

Zambia (21)	OPV	0, 6, 10, and 14 weeks, 12 months	18 months	133	397	Significantly lower antibody titer (difference in log_2_ antibody titer −0.62, 95% CI −1.04; −0.21, *p* < 0.01)

South Africa (25)	DTP-Hib or DTaP-IPV/Hib, HBV, PCV	6, 10, and 14 weeks	Birth, 16 weeks	38	55	Pre-vaccination: significantly lower antibody concentrations to Hib, pertussis, pneumococcus, and tetanus; lower % protected against Hib (17 vs 52%, *p* < 0.001), pertussis (24 vs 57%, *p* = 0.001), tetanus (43 vs 74%, *p* = 0.002), and hepatitis B (21 vs 54%,*p* = 0.01) Post-vaccination: following 1–2 doses, higher antibody concentration against Hib (6.46 vs 0.52 mg/L, *p* = 0.02), pertussis (81.16 vs 11.6 FDA IU/mL, *p* < 0.001), pneumococcus and tetanus (1.86 vs 0.50 IU/mL, *p* = 0.01). Following 3 doses, higher antibody concentration against pertussis (270.1 vs 91.7 FDA U/mL, *p* = 0.006) and pneumococcus (47.32 vs 14.77 mg/L, *p* = 0.001) Greater fold increase in antibody level against Hib (21.15 vs 2.97, *p* = 0.007) and pertussis (9.51 vs 2.16, *p* = 0.007) Infant:maternal antibody ratio (proxy for placental transfer of antibody) lower by 23% for Hib, 40% for pertussis, and 27% for tetanus in HIV-infected compared with HIV-uninfected mothers

South Africa (33)	DTP, Hib, HBV, measles	6, 10, and 14 weeks; DTP booster 18 months; measles 9, 18 months	0.5, 1.5, 3, 6, 12, 18, and 24 months	27	28	Pre-vaccination, significantly lower antibody levels against tetanus (*p* < 0.025) and higher against hepatitis B (*p* < 0.025) DTP, Hib, and HBV: after 2 doses, no difference in antibody levels or % protected. After 3 doses: higher antibody level and % protected against pertussis. At 24 months higher antibody level against tetanus *Measles*: no differences between groups

Denmark (34)	Hib	3, 5, and 12 months	15 months	19	7	No difference in antibody concentration

Brazil (35)	HBV, DTP/Hib	HBV: 0, 1, and 6 months DTP/Hib: 2, 4, and 6 months	7 months	53	112	HBV: more non-responders (6.7 vs 3.6%, χ^2^ 10.93, df = 1) and more very good responders (64.4 vs 38.8%, non-significant) among HIV-exposed infants Tetanus: significantly lower antibody titer against tetanus (GMT 1.520 vs 2.712, *p* = 0.013), but 100% of infants were protected Diphtheria: no significant differences between groups

*M. tb, Mycobacterium tuberculosis; OPV, oral polio vaccine; PCV, pneumococcal conjugate vaccine; DTwP-Hib, diphtheria, tetanus, whole cell pertussis, Hib; HBV, hepatitis B vaccine; DTaP-IPV/Hib, diphtheria, tetanus toxoid, and acellular pertussis combined with inactivated polio vaccine and Hib; HBsAg, hepatitis B surface antigen; % protected, proportion protected; GMC, geometric mean concentration; GMT, geometric mean titer; HIV, human immunodeficiency virus; Ig, immunoglobulin; CI, confidence interval*.

After three doses of pertussis-containing vaccine, antibody levels in HIV-exposed, uninfected infants were two to seven times higher than in unexposed infants (9, 29, 37). However, following the booster dose at 18 months, the proportion of children with protective antibody levels was non-significantly higher in HIV-exposed, uninfected infants than in HIV-unexposed children (37).

Following the first one to two doses of Hib vaccine, concentration of specific antibody was 12 times higher at 16 weeks in HIV-exposed, uninfected than unexposed infants (29), but there was no significant difference when infants had received all three doses (9, 29, 37, 38). Similarly, tetanus antibody concentration was higher after one to two doses, but not significantly different at 4, 5, or 6 months following three doses (9, 29, 37). Six months after the fourth (booster) dose, at 24 months, the antibody concentration was significantly higher in the HIV-exposed, uninfected children than in the HIV-unexposed children (*p* < 0.05) (37). One study found that at 7 months tetanus antibody concentration was significantly lower in HIV-exposed, uninfected infants than unexposed infants, but neither study found any significant difference in the proportion of infants protected (37, 39).

In studies in South Africa, HIV-exposed, uninfected infants who received all three doses of PCV had significantly higher antibody concentrations at 16 and 20 weeks (7, 29), although opsonophagocytic activity was reduced compared with unexposed infants for 1 out of 3 serotypes (7).

The increased antibody responses to the initial doses of Hib, tetanus, and other vaccines can be explained by reduced interference from maternally derived antibody in HIV-exposed, uninfected infants. Before vaccination, HIV-exposed, uninfected infants consistently had lower antibody concentrations against PCV (seven serotypes), pertussis, Hib, and tetanus in a number of studies (8, 9, 29, 37). For each of these specific antibodies, there was significantly reduced placental transfer, with reductions of 15–40% in the ratio of maternal antibody to infant antibody concentrations at birth (29). Individual infants with lower antibody levels at birth had larger antibody responses at 16 weeks, and HIV-exposed, uninfected infants had a significantly larger fold increase than unexposed infants following vaccination against PCV, pertussis, and Hib (29).

Two South African studies have compared measles vaccine responses in HIV-exposed, uninfected, and unexposed infants. One study found antibody concentrations were 36% higher at 16 months, but after the booster dose, both the antibody titer and proportion of infants protected were lower at 24 months (79.6 vs 94.3% protected, *p* = 0.002) (10). The other study found the opposite; however, in this study antibody responses were lower in all groups, especially HIV-unexposed infants, for whom only 50% had antibody concentrations associated with protection at 2 years (37). Pre-vaccination levels of measles antibody did not differ significantly in HIV-exposed and unexposed infants in either study (10, 37).

Antibody responses to hepatitis B vaccine in HIV-exposed, uninfected infants were heterogeneous, with higher proportions of both non-responders (6.7 vs 3.6%, χ^2^ 10.93, df = 1) and very good responders (64.4 vs 38.4%, non-significant) at 7 months, compared with HIV-unexposed infants in Brazil (39). At time points between 3 and 24 months, no significant differences were found in the overall proportion of infants with protective antibody levels in HIV-exposed, uninfected, and unexposed groups in Brazil and South Africa (9, 29, 37, 39). Before vaccination, the proportion of infants with protective antibody levels was higher in HIV-exposed than unexposed infants in two studies from South Africa and lower in one study in the same country (9, 29, 37).

In two studies of responses to diphtheria vaccine, antibody responses in HIV-exposed, uninfected, and HIV-unexposed infants did not differ, with more than 98% protected following the primary course (9, 39). Pre-vaccination anti-diphtheria toxin antibody levels were significantly higher in HIV-exposed, uninfected infants (GMC 0.136 vs 0.078, *p* < 0.001) (9). There were no differences found in IgG concentrations against OPV at 10 weeks (13). The response to OPV at 18 months was lower in HIV-exposed uninfected infants than unexposed infants, but this was no longer significant after adjusting for breastfeeding duration (26).

In summary, typically vaccines for which the antibody concentration is lower before vaccination result in higher concentrations after vaccination. This is true for only the first one to two doses of Hib and tetanus vaccines, but persists to the end of the course of PCV and pertussis. For all four vaccines there is reduced maternal trans-placental transfer of antibody (29). There are less clear trends for measles and hepatitis B vaccines, which may be more dependent on population transmission and prevalence. HIV exposure did not appear to have any effect on antibody responses to diphtheria or OPV. Overall, HIV-exposed, uninfected infants respond at least as well to vaccines as their unexposed peers.

### Cellular Responses

Human immunodeficiency virus-exposed, uninfected infants produce strong T-cell responses to BCG vaccine. In South Africa, BCG-specific CD4 and CD8 T-cell proliferation increased significantly after vaccination in HIV-exposed, uninfected, and unexposed infants at 14 weeks (40). In another study, all 94 HIV-exposed, uninfected infants formed a scar (41). T-cell proliferation and cytokine secretion were not affected by maternal HIV infection or *Mycobacterium tuberculosis* (*M. tb*) sensitization at time points between 6 weeks and 12 months (13, 27, 40, 42, 43).

Differences in the frequencies of specific T-cell subpopulations have been found between HIV-exposed, uninfected, and unexposed infants before and after BCG vaccination (40, 42). In HIV-exposed and uninfected infants, the CD4 and CD8 T-cell response at 14 weeks was less polyfunctional, indicating a less effective response (42). However, this may simply reflect immaturity, as infants were vaccinated within 3 days after birth, and another study in which the infants were vaccinated at 6 weeks found very little difference in T-cell subpopulations at 16 weeks, compared with HIV-unexposed infants (40).

At birth, no differences in BCG-specific T-cell proliferation or functionality are seen between HIV-exposed, uninfected, and unexposed infants (40). However, there are differences in the frequencies of some T-cell subsets, some of which correlate between mother-infant pairs, with the strongest associations between HIV-infected, *M. tb* sensitized mothers, and their infants (40). Secretion of TNF-α and IFN-γ in response to BCG antigens was increased at birth in HIV-exposed uninfected infants compared with HIV-unexposed infants, but only when their mothers had evidence of latent tuberculosis infection (40). These findings support the idea that HIV-exposed uninfected infants are able to mount just as robust a response to BCG vaccine as unexposed infants, but that the immune system may be primed by antenatal exposure to maternal HIV and tuberculosis infection (40).

Two studies have investigated the cellular response to other vaccine antigens in HIV-exposed, uninfected infants compared with unexposed infants. In response to pertussis vaccine, one study found no significant differences in T-cell proliferation at 14 weeks in HIV-exposed, uninfected, and unexposed infants, but HIV-exposed infants showed reduced polyfunctionality in CD4 and CD8 responses (42). Similarly, tetanus vaccine-specific T-cell responses showed no differences at 3 months, but at 12 months HIV-exposed uninfected infants had reduced polyfunctionality, and a lower proportion of effector memory T-cells compared with HIV-uninfected infants (43).

A similar pattern was seen in response to stimulation with staphylococcal enterotoxin B (SEB) in one study, even after adjusting for differences in birthweight, breastfeeding, and gestational age (42). However, another study found that cytokine production and polyfunctionality were increased overall at 3 months but reduced at 12 months (43).

### Mechanisms of Altered Vaccine Responses in HIV-Exposed, Uninfected Infants

Human immunodeficiency virus-exposed, uninfected infants are exposed to antenatal factors that might affect both their antibody and T-cell responses to vaccines. There is compelling evidence that in mothers with HIV infection, less IgG is transferred across the placenta than in HIV-uninfected mothers, resulting in lower pre-vaccination levels of IgG specific to several vaccines (8–10, 29, 37). Results from analysis adjusting for maternal age, gravidity, and socioeconomic status show that maternal HIV infection is associated with the concentration of specific IgG following Hib, pertussis, PCV, and tetanus vaccines in exposed, uninfected infants (26, 29). In this study, mothers received ART during and after pregnancy, infants received zidovudine for the first month after birth, and no HIV-exposed, uninfected infants were exclusively breastfed. This finding is likely to be a result of lower vaccine-specific antibody levels in HIV-infected mothers, which correlate with CD4 count (29), and placental dysfunction resulting in reduced placental transfer of antibody (8, 9, 29, 37). Maternally derived antibody present in infants pre-vaccination may inhibit the infants’ own IgG responses, leading to the observation that infants with the highest pre-vaccine levels of anti-body had the lowest fold increase following vaccination (29). Although the mechanisms for this are incompletely understood in humans, animal models have shown that this inhibition is mediated by maternally derived antibody binding to vaccine antigens, which then form cross-linkage between the B cell receptor (which binds vaccine antigen) and the FcγIIB receptor (which recognizes the Fc portion of IgG). This results in inhibitory signals, reduced proliferation of B cells and decreased secretion of vaccine-specific IgG (44, 45).

Infants of mothers with HIV infection may be exposed antenatally to HIV proteins and/or maternal immune factors that have a wider effect on the development of the immune system *in utero* and early infancy. HIV-exposed, uninfected infants may have a smaller thymus, which has been associated with immune abnormalities in early infancy (38). There is some evidence that T-cells in HIV-exposed, uninfected infants show changes in proliferation and phenotype compared with HIV-unexposed infants (13). CD4 count may be significantly lower and represent a smaller proportion of total lymphocytes, and some studies have found an association between infant and maternal CD4 count (13, 46). The reduction in T-lymphocytes occurs mainly in less differentiated subsets, and cells expressing markers of replicative senescence (CD57 and PD-1) are more frequent (13). At birth and 6 weeks, the background concentration of IFNγ was reported to be significantly higher in HIV-exposed, uninfected infants than unexposed infants in one study in South Africa (41). These early changes could represent priming of some aspects of the immune response *in utero*, leading the alterations in proliferation and function of T-cell subsets in response to vaccinations in HIV-exposed infants (13, 39–41).

Another antenatal factor that may affect HIV-exposed, uninfected infants is exposure to ART. Nevirapine has been associated with slightly increased markers of immune activation in cord blood (47), and maternal ART was associated with reduced neutrophil and lymphocyte counts in HIV-exposed, uninfected infants, with the largest difference in infants of mothers on combination therapy (46). However, an association between maternal ART and infant vaccine-specific T-cell responses has not so far been demonstrated (42, 43).

Increasing maternal age is associated with higher infant levels of pertussis antibody at birth (29). This might be influenced by differing maternal exposure to circulating pertussis or to different vaccine coverage with pertussis vaccines at different times. Other maternal infections during pregnancy are likely to be important in determining infant antibody concentrations pre-vaccination and therefore potentially post-vaccination too, for example, high variability in infant hepatitis B antibody response is likely to be a result of higher prevalence of hepatitis B infection in HIV-infected mothers in some settings (37).

Postnatally both maternal and environmental factors probably have important effects on infant vaccine responses. Breastfeeding is an important conduit for transfer of IgA from mother to infant and is associated with larger thymic size, phenotypic changes to lymphocyte subpopulations and improved immune function (48). In studies of HIV-exposed, uninfected, and unexposed infants, there are often large differences in breastfeeding practices between groups (26, 29, 37), and many studies do not report data on breastfeeding (7–10, 13, 38–40, 43). One study reported that the reduction in neutralizing antibody response to OPV in HIV-exposed, uninfected infants could be accounted for by reduced breastfeeding duration (26). This could be because of reduced antibody transfer, or increased exposure to maternal infections such as CMV which are transmitted in breast milk (26).

Postnatal exposure to other infections may also affect specific antibody responses to vaccines. The large differences between studies in the proportion of infants protected against measles following vaccination raises the possibility that differences in transmission rates of measles infection may have affected the proportions of infants protected (49). Differences in nasopharyngeal colonization with pneumococcus among HIV-exposed, uninfected infants, and unexposed infants has also been suggested to contribute to differences in their vaccine responses (7). In low-resource settings HIV-exposed, uninfected infants have poorer nutritional status than unexposed infants (50), although a trial of nutritional supplementation between age 6 and 18 months had no effect on antibody responses to OPV in HIV-exposed, uninfected infants (26).

We conclude that there is convincing evidence that reduced antibody transfer across the placenta is associated with changes in antibody responses to the initial doses of PCV, tetanus, pertussis, and Hib vaccines in HIV-exposed, uninfected infants. The effect of HIV exposure on responses to hepatitis B and measles vaccines appears more variable between populations, and prevalence of these infections may be an important factor. The functional quality of vaccine-specific antibody produced by HIV-exposed, uninfected infants requires further investigation (29, 37), but overall it is encouraging that HIV-exposed, uninfected infants do not appear to have significantly reduced levels of protection from routine infant vaccines. Breastfeeding is likely to affect responses to other vaccines besides OPV, and further studies are now more feasible following changes in WHO recommendations to support breastfeeding in HIV-infected mothers in a wider range of settings (51).

## Vaccine Responses in Infants with Congenital and Postnatal CMV Infection

### Effect of CMV Infection on T-Cell Populations

Congenital and postnatal CMV infection leads to a series of changes in infant CMV-specific CD4 and CD8 T-cells, as well as having an effect on the whole T-cell population. There is an initial increase in activation of the whole CD8 T-cell population, which returns to normal over 12–24 months (52–55). CMV-specific CD8 T-cells remain highly activated for at least 24 months following postnatal infection, but in congenital infection, activation may diminish more rapidly (53, 54). There is a shift toward more differentiated CD4 T-cells, but CMV-specific CD4 T-cells are infrequently found in infected infants (55, 56). In adults with CMV, these cells are common and are associated with effective control of viral replication, less severe disease, and lower risk of mother-to-child transmission (57, 58).Infant T-cells are less polyfunctional than those seen in adults, and polyfunctionality is also thought to be associated with improved control of CMV infection (55, 58–60). Therefore, congenital and postnatal CMV infection affects the whole T-cell population, and the effect is different in infants compared with adults. Infants have a longer duration of viremia than adults (55, 61), and their vaccine responses may be affected differently by CMV infection.

### Effect of CMV Infection on Humoral and Cellular Vaccine Responses

Studies in elderly adults have shown that latent CMV infection leads to the expansion of CD8^+^CD28^−^ T-cells, which are thought to suppress immune responses to influenza vaccine and contribute to generalized immunosenescence in older adults (14). Few studies have evaluated the effects of CMV infection on infant vaccine responses (Table 3).

**Table 3:** Effect of CMV infection on infant responses to vaccines.

	Vaccine	Age at vaccination	Age at blood sampling	CMV-infected infants, *n*	CMV-uninfected infants, *n*	Findings
Gambia (51)	Measles; tetanus, Hib	9 months; 2, 3, 4, and 16 months	9 months	86	46	CMV-infected vs uninfected infants (congenitally and postnatally infected infants in same cohort): Infected infants had lower CD4 IFN-γ response to measles (*p* = 0.013) No significant difference in CD8 T cell proliferation or IFN-γ response to measles No difference in measles antibody titers Infected infants’ IFN-γ response to CMV correlated with measles antibody response at 13 months No significant difference in IgG response to Hib or tetanus vaccines at 18 months
			13 months	90	42	
			18 months	121	11	

Gambia (59)	Measles, meningococcus A and C	9 months	Birth	0	224	Comparison of CMV and EBV singly infected, co-infected, and uninfected infants CMV status had no significant effect on measles antibody titer Infection with EBV reduced measles antibody response, except when there was co-infection with CMV (median log_2_ hemagglutinin antibody inhibition assay titer EBV^+^CMV^−^ = 3.0, EBV^+^CMV^+^ = 5.0, *p* = 0.003) CMV status had no significant effect on anti-meningococcus IgM or IgG
			9 months	115	58	
			11 months	121	51	

Zambia (21)	Oral polio	Birth, 6, 10, and 14 weeks, 12 months	18 months	369	75	No significant associations between IgG response to OPV and CMV infection. In CMV seropositive infants, % vaccine failure was slightly lower than in seronegative infants (1.4 vs 4.0%, *p* = 0.14) In HIV^+^ infants, Ab titers were lower in infants with CMV viremia than without (log_2_ antibody titer 3.2 vs 5.75, *p* = 0.14) In HIV-unexposed infants, Ab titers were higher in CMV seropositive infants (log_2_ antibody titer 8.31 vs 7.77, *p* = 0.11) In HIV-exposed uninfected infants, CMV had no significant effect

*EBV, Epstein–Barr virus; Ig, immunoglobulin; HIV, human immunodeficiency virus; CMV, cytomegalovirus; OPV, oral polio vaccine; IFN, interferon*.

In a study of measles vaccine in Gambian infants, 1 week after vaccination infant CD8 T-cell responses did not vary with CMV infection acquired congenitally or postnatally, but CD4 T-cell IFN-γ responses were lower in CMV-infected infants than in infants without CMV infection (54). At 13 months of age, there were no differences in memory T-cell responses between CMV-infected and uninfected infants. However, CMV-infected infants showed significantly higher CD4 and CD8 T-cell IFN-γ responses to SEB, indicating that immune activation is present in CMV-infected infants. Furthermore, there was a positive correlation between the magnitudes of the responses to SEB and CMV (54).

A study of CMV and EBV co-infection supports the idea that the effect of CMV infection on antibody responses to measles is dependent on changes to the T-cell population. Epstein–Barr virus (EBV) infects B-cells, and EBV-infected infant IgG responses to measles vaccine and meningococcus A and C polysaccharide vaccine are reduced by approximately one third (62). In infants co-infected with CMV and EBV, the measles-specific IgG vaccine response is similar to uninfected infants. However, CMV co-infection does not have a significant effect on the IgG response to meningococcus (a T-cell independent response) in EBV-infected infants, and the vaccine response is still lower than in EBV uninfected infants (62).

Gambian infants who acquired CMV antenatal or postnatally had no significant differences in anti-Hib or anti-tetanus toxoid IgG concentration measured compared with uninfected infants at 18 months (54). A study of antibody response to OPV in Zambian infants found that neither CMV seropositivity nor viremia had a significant effect on OPV neutralizing antibody titers or frequency of vaccine failure at 18 months of age (26). However, trends in the data suggested that co-infection with HIV and CMV may have negative synergistic effects on the antibody response to OPV. CMV seropositivity at 18 months was associated with a trend toward a small decrease in vaccine failures in HIV-unexposed infants (0.4 vs 4.3% vaccine failure, *p* = 0.06), but not in HIV-exposed, uninfected infants. In HIV-infected infants antibody responses were reduced in CMV seropositive compared with CMV seronegative infants, although these results did not reach statistical significance (26).

Human immunodeficiency virus-infected mothers in this study had a mean breastfeeding duration of 6 months compared with 15 months in HIV-uninfected mothers, and longer breastfeeding duration was associated with increased mean poliovirus antibody titers in infants (26). The authors do not state whether mean breastfeeding duration differed between CMV seropositive and seronegative groups, but this is important because breastfeeding is the main route of transmission of postnatal CMV infection, and in HIV-exposed, uninfected infants in this study, the reduction in neutralizing antibody response to OPV could be accounted for by reduced breastfeeding duration (26).

Cytomegalovirus-infected infants show alterations in responses to measles and possibly polio vaccines, which are live vaccines, and no significant differences have been found in the responses to Hib or tetanus vaccines. Overall the ability to mount effective and lasting responses is preserved in otherwise well infants, at least in the short term, and responses to SEB indicate that some T-cell responses are increased in CMV infection. Interactions may occur between CMV and HIV or EBV to produce further alterations in vaccine responses, but there is still no significant impairment compared with CMV-uninfected infants. There is limited data on the effects of CMV infection on infant vaccine responses, and in light of growing evidence of poor clinical outcomes associated with CMV infection in HIV-exposed, uninfected infants, further studies are particularly important in this group.

## Conclusion

Human immunodeficiency virus-infected infants have some impairment in their humoral and cellular responses to routine immunizations. However, as many of the infants in the studies reviewed were born to mothers who started ART a short time before delivery as part of PMTCT programs, and were not exclusively breastfed, future studies will be needed to determine whether the same changes in immune responses are present when mothers and infants undertake optimal HIV treatment, PMTCT, and feeding practices. The clinical importance of these findings is unknown, as the risk of vaccine-preventable infection in HIV-infected infants compared with HIV-unexposed infants has not been determined.

Human immunodeficiency virus-exposed, uninfected infants and those with CMV have alterations in their vaccine responses, but the evidence does not support changes to the vaccine schedule in these groups. Protecting infants from infection before their first vaccines, for example, by maternal immunization, is important in all infants, even more so in HIV-exposed, uninfected, and HIV-infected infants, who are less likely to be protected than HIV-unexposed infants. Maternal immunization is a key part of global efforts to reduce neonatal and infant infectious morbidity and mortality. A better understanding of the mechanisms by which maternal infection and immune responses influence the developing infant immune system are critical to ensuring the success of new vaccines.

## Author Contributions

The theme and concept were designed by M-LN and CJ. OF undertook the literature review and wrote the original draft of the paper, which was reviewed and revised by CJ and M-LN.

## Conflict of Interest Statement

The authors declare that the research was conducted in the absence of any commercial or financial relationships that could be construed as a potential conflict of interest.
