# Supplementary material for: The Effect of Human Immunodeficiency Virus and Cytomegalovirus Infection on Infant Responses to Vaccines: A Review
Source: Front Immunol. 2018 Mar 2;9:328. doi: 10.3389/fimmu.2018.00328 (PMC5840164; doi:10.3389/fimmu.2018.00328)
Supplement: Supplementary file 1 [file Table_1.docx]

**Supplementary material**

**Table 1: search strategy**

Maternal HIV

| 1. | Title or abstract | Mother* or maternal or pregnan* or antenatal or utero or uterine |
| --- | --- | --- |
|  | Or MeSH terms | Pregnancy Complications, Infectious or Maternal-Fetal Exchange or Infectious Disease Transmission, Vertical |
| 2. | Title or abstract | HIV |
|  | Or MeSH terms | HIV infections/im [immunology] or HIV infections/co [complications] or HIV seronegativity |
| 3. | Title or abstract | neonate* or neonatal or infant* or child* or newborn* or f?etus or f?etal or perinatal* |
|  | Or MeSH terms | Infant or Child or Fetus |
| 4. | Title or abstract | (humoral adj response*) or (vaccine adj2 response*) (antibody adj response*) or (immune adj response) or immunogenicity or immunoglobulin* or antibod* or T-lymphocyte* |
|  | Or MeSH terms | Antibodies, bacterial or Antibodies, viral or Antibodies, neutralizing or Immunologic Memory or Immunoglobulin G or Immunoglobulin M or Immunophenotyping or Lymphocyte subsets or T-lymphocytes or Immunity, cellular or Immunity, humoral |
| 5. | Title or abstract | Vaccin* or immuni?e* or immuni?ation* or BCG or tuberculosis or hepatitis B or polio or diphtheria or tetanus or pertussis or DTP or haemophilus or Hib or pneumococcal or rotavirus or MMR or measles or mumps or rubella or meningococcal or varicella or chickenpox or influenza |
|  | Or MeSH terms | Vaccination or Vaccines or Immunization schedule or Immunization Programs or Immunization or Pneumococcal Vaccines or Rotavirus Vaccines or Diphtheria Vaccines or Tetanus toxoid or Diphtheria-Tetanus-Pertussis Vaccine or Tuberculosis Vaccines or BCG Vaccine or Viral Hepatitis Vaccines or Hepatitis B Vaccines or Meningococcal Vaccines or Haemophilus Vaccines or Measles Vaccine or Mumps Vaccine or Rubella Vaccine or Measles-Mumps-Rubella Vaccines or Chickenpox Vaccine or Poliovirus Vaccines or Influenza vaccines |
| 6. |  | 1 and 2 and 3 and 4 and 5 |
| 7. |  | 6 limited to Humans, English |
| 8. | Publication type | Not Letter or editorial or news or review |

Maternal CMV

| 1. | Title or abstract | Infant* or newborn* or neonate* or neonatal* or antenatal* or utero or uterine or f?etus or f?etal or perinatal* or neonatal* or congenital* |
| --- | --- | --- |
|  | Or MeSH terms | Infant or Infant, Newborn or Fetus/im, vi [immunology, virology] |
| 2. | Title or abstract | Cytomegalovirus or CMV |
|  | Or MeSH terms | Cytomegalovirus infections |
| 3. | Title or abstract | antibod* or t-lymphocyte or (immune response*) |
|  | Or MeSH terms | Immunity, Cellular or Immunity, Humoral or Immunologic Memory or Antibodies or T-lymphocyte Subsets |
| 4. | Title or abstract | Vaccin* or immuni?e* or immuni?ation* or BCG or tuberculosis or hepatitis B or polio or diphtheria or tetanus or pertussis or DTP or haemophilus or Hib or pneumococcal or rotavirus or MMR or measles or mumps or rubella or meningococcal or varicella or chickenpox or influenza |
|  | Or MeSH terms | Vaccination or Vaccines or Immunization schedule or Immunization Programs or Immunization or Pneumococcal Vaccines or Rotavirus Vaccines or Diphtheria Vaccines or Tetanus toxoid or Diphtheria-Tetanus-Pertussis Vaccine or Tuberculosis Vaccines or BCG Vaccine or Viral Hepatitis Vaccines or Hepatitis B Vaccines or Meningococcal Vaccines or Haemophilus Vaccines or Measles Vaccine or Mumps Vaccine or Rubella Vaccine or Measles-Mumps-Rubella Vaccines or Chickenpox Vaccine or Poliovirus Vaccines or Influenza vaccines |
| 5. |  | 1 and 2 and 3 and 4 |
| 6. |  | 5 limited to Humans, English |
